# Empirical radio propagation model for DTV applied to non-homogeneous paths and different climates using machine learning techniques

**DOI:** 10.1371/journal.pone.0194511

**Published:** 2018-03-29

**Authors:** Igor Ruiz Gomes, Cristiane Ruiz Gomes, Herminio Simões Gomes, Gervásio Protásio dos Santos Cavalcante

**Affiliations:** 1 Department of Computation, Federal University of Pará, Belém, Pará, Brazil; 2 Department of Mathematics, Federal University of Pará, Belém, Pará, Brazil; 3 Department of Telecommunications, Federal University of Pará, Belém, Pará, Brazil; SPAIN

## Abstract

The establishment and improvement of transmission systems rely on models that take into account, (among other factors), the geographical features of the region, as these can lead to signal degradation. This is particularly important in Brazil, where there is a great diversity of scenery and climates. This article proposes an outdoor empirical radio propagation model for Ultra High Frequency (UHF) band, that estimates received power values that can be applied to non-homogeneous paths and different climates, this last being of an innovative character for the UHF band. Different artificial intelligence techniques were chosen on a theoretical and computational basis and made it possible to introduce, organize and describe quantitative and qualitative data quickly and efficiently, and thus determine the received power in a wide range of settings and climates. The proposed model was applied to a city in the Amazon region with heterogeneous paths, wooded urban areas and fractions of freshwater among other factors. Measurement campaigns were conducted to obtain data signals from two digital TV stations in the metropolitan area of the city of Belém, in the State of Pará, to design, compare and validate the model. The results are consistent since the model shows a clear difference between the two seasons of the studied year and small RMS errors in all the cases studied.

## 1 Introduction

Digital television systems are designed to ensure the received signal for the users is of a high standard. However, it is not enough to define the parameters that maintain the good quality of the signal, and studies are needed to analyze the propagation loss between the transmitter and receiver. Radio propagation models are an efficient way of analyzing and predicting the signal strength. The signal suffers degradation along the propagation path due to several factors such as reflection, diffraction, absorption, scattering.

The diversity and complexity of scenarios in urban areas has led to the development of a number of propagation models that are suited to these environments. Empirical and deterministic propagation models that allow corrections/modifications for different types of terrain morphology have been discussed in recent years. However these models usually only apply building criteria [1, 2] or vegetation criteria [3, 4] or sea paths [5–7] or even paths between rivers and lakes [8, 9] on an individual basis.

One of the most widely used international recommendations is for terrestrial paths with frequency range of 30 to 3000 MHz is ITU-R P.1546-4. This consists of interpolations and extrapolations of distance, frequency, the height of transmitting antennas and percentage of time. It includes corrections for the effective height of the receiving antenna, terrain clearance angle, among other factors. There are also tables for different kinds of terrestrial paths, warm sea, cold sea and coastal region paths for the propagation models, although they do not include the case of fresh water. The most commonly accepted approach for this kind of path is to treat entirely overland. However, this can only be acceptable for small stretches of freshwater but not for rivers of a significant size such as those found in the Amazon region. No account was taken of different climatic conditions.

An empirical propagation model that allows transitions between different morphologies in urban areas is proposed in [1]. It was observed that there was an increase of received power in the boundaries (owing to the recovery effect).

Two deterministic models for forest environment are examined in [3, 4]. A parabolic equation (PE) is shown in [3]. The results were compared with measurement data from Amazonian towns and cities and also with other models. The main advantages of PE are the reduction of computational time compared with the similar techniques found in the literature and the unconditional stability that allows changes to be made in the parameters of other scenarios. A model for foliage attenuation at 3.5 GHz in German rural areas is shown in [4]. The authors have designed an analytical model that replaces the canopy of a tree in a discrete random medium with statistical features that are related to the physical quantities of the tree.

Articles [5, 8, 9] include reviews, comparisons and modifications of ITU-R P.1546-4. In [8, 9] some changes are recommended for land-freshwater paths. An empirical approach that employs reverse interpolation is adopted in [8] and a deterministic approach using the 1st Fresnel ellipsoid can be found in [9]. In both cases it was confirmed that in the recommendation, the prediction was underestimated.

This paper puts forward an empirical radio propagation model for DTV for non-homogeneous paths and different climates based on machine learning techniques. The proposed model has two innovative features: i) its application in non-homogeneous paths includes long stretches of fresh water; ii) it distinguishes between the seasons. These two factors have been poorly studied in the UHF range, as long stretches of fresh water are common in towns and cities located in equatorial/tropical forests. The model set out here is a hybrid based on two machine learning techniques K-Nearest Neighbors (KNN) and Knowledge-based theory (KBT), that allow different attributes (both quantitative and qualitative) to be treated in a clear and efficient way. Data from a measurement campaign carried out in Belém city located in the Amazon estuary was used for purposes of comparison and to validate the model.

## 2 Related works

### 2.1 Propagation models for buildings

Propagation models that take account of buildings are the most common in the literature, and each of them make use of some particular feature of the environment or region of study, although some classic propagation models are used as base, such as the SUI, Okumura, Hata-COST 231, and COST Walfisch-Ikegami models.

These models rely on the distance, number of buildings, and classification of the region (urban, suburban, rural) to predict the signal strength.

Many articles draw on a range of these models to suit the particular features of the environment being studied. In [10] the authors carried out a measurement campaign in suburban areas of Brazil, and then compared their results with an extended Hata-COST 231 for frequencies under 6 GHz, SUI model and UFPA model (specifically designed for wooded areas in Brazil). The results show that the UFPA model (based on SUI) achieved the best prediction of signal strength.

### 2.2 Propagation models for vegetation

Analytical propagation models that take account of trees or forests have been discussed in the literature since the 1960s [11]. Further works have included a mixed terrain involving paths in both forests and treeless areas [12], models with three layers including a forest layer [13] and areas where there is a combination of forests and cities [14–16]; Others, even extend this to four layers: free space, a canopy of trees, trunks and forest soil [17, 18].

In [19] the authors employ a new methodology to design the guided propagation of radio waves. This methodology is called the Moving Window Finite Difference Time Domain (MWFDTD), which makes an improvement in classical FDTD because it assumes that a pulsed radio wave only exists in a small part of the propagation path at a given period of time. Moreover, it allows the use of a relatively small FDTD mesh which ensures that it only exists in the part of the region over which the pulse moves.

The vegetation only includes simple trees, or, else a single tree or trees scattered in the scenario—it does not include forested areas. The tree was designed as a dielectric rod with effective complex permittivity that depends on the height, orientation, density and constitutive parameters of the branches and leaves. The results obtained by the method were compared with experimental data obtained from the Institute for Telecommunication Sciences (ITS) in the frequencies of 230, 410 and 910 MHz. In all cases the results were regarded by the authors as satisfactory. There is no error in cases of comparison.

The recommendation of ITU-R P.833-9 [20] is to have several models to enable the reader to evaluate the effect of vegetation on radio wave signals. This recommendation also contains measured data of vegetation fade dynamics and delay spread characteristics. In the case of VHF and UHF, the recommendation does not take account of attenuation in a single tree, caused by specific attenuation that has relatively low values. For this reason, the length of the path and a specific attenuation are used for very short vegetative paths, although for dense and larger wooded areas in Brazil, the attenuation cannot be ignored.

In [21, 22] the authors presents a propagation model, adding the characteristics of the attenuation experienced by the radio wave when propagating in typical city environments of the Amazon region.

The received power measurements were collected from 335 fixed clients, spread across 12 cities in the northern region of Brazil. Mobility measurements were carried out on campus at the Federal University of Pará (UFPA). A comparison is also made between performance of the proposed model and that of other models (SUI and COST231-Hata) described in the literature for fixed and mobile wireless networks.

The model achieved a RMS error of 3.8 *dB* and standard deviation of 2.3 *dB*, which surpasses the other models that obtained RMS errors above 10 *dB* and standard deviations above 5 *dB*. The results obtained show that they are more effective than other models in predicting losses in the 5.8 GHz band in fixed and mobile systems.

A model for attenuation caused by foliage in the rural areas of Germany (3.5 GHz) is discussed in [4]. Several models for trees are examined, including one that uses cylinders for trunks and branches, and discs for leaves.

The results are compared with data measured in three seasons: winter, spring and summer. These authors found differences of 0.2 *dB*/*m* in the coefficient for effective foliage between the data for spring and summer. In addition, the attenuation in the spring is about 3.15 *dB* whereas in the summer it is 4.68 *dB*. These differences show the importance of studying, (at least in an indirect way), the environmental conditions of the path under study.

### 2.3 Propagation model for paths over water

The papers that are most common in the literature use overseas propagation, or long stretches of salt water.

Witvliet et al. [5] evaluated the ITU-R Recommendation P.1546-4 from a large amount of measured data and suggested some alterations. The data were measured in the Netherlands and the United Kingdom, by taking three different cities in the Netherlands as transmitters and three in the UK as receivers, making a total of 7 routes over mixed land with different percentages of sea routes. Data were collected from 8 different frequencies between 500 and 700 MHz. The authors collected as much as 21 million data in 500 days. They also described the data processing to provide a methodology for other measurement campaigns. Finally, the measured data were compared with the data predicted by the ITU-R model P.1546-4. In most cases there was an underestimation of the model with differences of up to 20 *dB*. The model based on the previous recommendation ITU-R P.370-7 [23] achieved considerably better results, which according to the authors was mainly due to the TCA correction factor in the receiver and the introduction of Δ*h* (treatment of small differences in heights), with a roughness of up to 50 *m*.

The terrestrial wave propagation model based on the asymptotic analysis method for HF in mixed terrain (land-sea) is examined in [24]. The performance of the model is compared with simulated and measured data. In both cases it performs well, and demonstrates the recovery effect at the land transition boundaries.

Mayrink et al. [9] make an estimation of the field intensity in the VHF and UHF bands for short runs (up to 10 *km*) partly over fresh water. The method is based on the intersection of the 1st Fresnel ellipsoid with the terrain profile. The terrain must be approximated by its equivalent plane, and the ellipsoid is defined so that the transmitting antenna can be located in one of the ellipsoid foci, while the image of the receiver is in the other focus. The main difference of this model from the ITU-R1546-2 [25] model is that it takes account of the percentage of water contained in the intersection of the ellipsoid with its flat equivalent distance and not its total amount.

Gomes et al. [8] employ an electric field prediction methodology for mixed ground- freshwater plots based on the ITU-R recommendation P.1546. This includes a case study with measurements in the metropolitan region of Belém-PA (Brazil), city that has a varied morphology ranging from densely urban perimeters to forest areas and rivers. The P.1546 recommendation was insufficient for the analysis of land and freshwater pathways in this scenario, both in their approach entirely over land and in the use of the correction for mixed plots. This methodology involves dividing the original problem into n minor problems, where n is the number of different lands crossed. The resulting n problems are treated in accordance with ITU-R Recommendation P.1546-4. The information about the previous lands is included in the calculation of the present lands, although the information of the subsequent lands is not taken into account. This is different from the one proposed in the recommendation that includes the information from all the lands. The methodology enables significant gains to be made in the modeling for mixed terrain-freshwater terrains, with a fall of RMS from 8.2 to 2.9.

### 2.4 Propagation models for different climatic conditions

There are many propagation models in the literature that provide information on rainfall rates, but most are intended for very high frequency bands such as the Ku and Ka bands for satellites. In these frequency bands, it is evident that there is a need for models that provide attenuation caused by rainfall, since the size of the rainwater droplet is considerable with regard to the wavelength.

This topic is particularly important because it shows the environmental influences and climatic condition in propagation.

In [26], a study is made of a radio link with a physical passage to the land-sea type on the Norwegian coast. The article shows that the climatic changes of the region interfere with the signal (e.g. rain and wet snow). The fading behavior is approximated to the ITU- R P.530-15 [27] model, but the model underpredicts the number of other parameters such as fading speed, enhancement, average fade duration, and events have been measured and compared with ITU-R P.530-15. The radio link activity has also been compared with the weather conditions at the time of the most severe fading incidents.

The works [28–30] include models for a correction of ITU-R recommendations P.618-9 [29], P.839-3 [31] and P.837-5 [32] for rainfall rates in tropical countries, Singapore and Malaysia, respectively.

The work of Meng, Lee and Ng [33–35] studies the influence of rainfall and wind on the propagation of waves in the UHF and VHF range in forest areas. In all three studies, the authors used campaign data from measurements made at a palm plantation in Singapore, where the trees are equally spaced and have an average height of 5.6 *m* and trunk diameter of 0.4 *m*.

In Meng, Lee and Ng [33], the authors provide evidence that the lateral wave is dominant in the propagation within the UHF band and is not influenced by the presence of rainfall, and is thus able to propagate under the canopy of trees in a similar way to free space. On the other hand, the multistage component induced by the spreading on the leaves and branches, is significantly affected by the intensity of the rain. This is mainly due to the increase of water in the foliage which leads to greater attenuation and absorption of the multi-component. The authors found that there is an increase in RMS and number of multipath clusters, as the rain subsides.

Meng, Lee and Ng [34] carry out a statistical study of the loss of propagation caused by rain and wind. The authors analyze several situations involving rainfall and weak, moderate and strong winds and combinations of these. An increase of the attenuation was observed when there is an increase of rain, but the most significant fact was the intensity of the wind because of the extent to which it altered the distribution of the leaves in the type of tree studied.

Meng, Lee and Ng [35] carried out a study of a 4-layer model for a transmitter and receiver within the forest. This allowed the authors to make a comparison with traditional models, and showed that models like COST231 and ITU-R fail to achieve good results in wooded regions.

## 3 Machine learning techniques

Machine Learning (ML) is a sub-area of Artificial Intelligence (AI) that is designed to create algorithms or develop methods that allow the computer to learn, or find patterns in a dataset.

According to [36] there are three types of learning: supervised, unsupervised and reinforcement. Alpaydin [37] divides machine learning into the following: Learning associations or knowledge-based theory (KBT) (using the deductive method); Classification; Regression; Unsupervised learning and reinforcement learning.

The supervised learning determines a mapping of inputs *x* and outputs *y*, given a set of pairs $D={\left\{\left(x_{i},y_{i}\right)\right\}}_{i=1}^{N}$ called the training set and N the number of elements of this set. Each component of the p-dimensional *x*_*i*_ vector is called an attribute. When the output *y*_*i*_ consists of qualitative information, the problem is known as the classification or recognition of standards, if *y*_*i*_ is formed of real numbers, the problem is known as regression.

Unsupervised learning aims at determining patterns that are of interest when there is only input data $D={\left\{x_{i}\right\}}_{i=1}^{N}$. This type of problem is much less well-defined since we do not know what patterns to fetch and there is no obvious metric for error calculation [38].

### 3.1 Knowledge-based theory (KBT)

Knowledge-based agents use the deductive logical method; they are mathematical models based on Facts and Rules. For [39], “A knowledge-based agent comprises a knowledge base and an inference engine”.

Facts are a priori knowledge obtained by the observation of a phenomenon. These can be registered qualitatively (empirically) by a specialist or quantitatively by a device, thus forming a Knowledge Base (KB).

Rules are logical combinations of facts. This feature is often used in an IF-THEN format, the first part known as predecessor (or premise) and the second part as due (or conclusion). One can also add multiple antecedents united by conjunction or disjunction (and/or) [40].

Rules can be strong or weak. A strong rule assigns a high level of certainty to the facts, otherwise the rule is said to be weak. The strength of each rule is usually given by a real number belonging to the interval [0, 1] called the weight. The rules are assigned to ensure the sum of the weights of all the rules equals 1 for a given fact. New rules can be obtained in the process through the combination of rules and initial facts, and this process is called inference.

The robustness of a knowledge-based model is linked to the number of facts observed and the quality of the rules that are applied. Therefore, the more facts there are, the more rules can be drawn up for a better description of the phenomenon.

### 3.2 K-Nearest Neighbors classifier

K-Nearest Neighbors (KNN) is a classifier where learning is based on analogy. The training set is formed of n-dimensional vectors and each element of this set represents a point in n-dimensional space.

When determining the class of an element that does not belong to the training set, the KNN classifier looks for the *K* elements of the training set that are closer to the unknown element, that is to say, they have the smallest “distance”. The class of *K* elements closest to the unknown element is verified and the same class is assigned to it. There is a risk of over training in all types of supervised learning. When over training occurs the analysis possibly contains noises besides the main signal, which can lead to a misinterpretation of the results, so it becomes necessary to review a specialist to analyze the results.

The most common metrics for determining the distance between two points *X* = (*x*_1_, *x*_2_, …, *x*_*n*_) and *Y* = (*y*_1_, *y*_2_, …, *y*_*n*_) of ℜ^*n*^ are:

- Euclidian Distance
  $$\begin{array}{c} d\left(X,Y\right)=\sqrt{{\left(x_{1}-y_{1}\right)}^{2}+{\left(x_{2}-y_{2}\right)}^{2}+\ldots +{\left(x_{n}-y_{n}\right)}^{2}} \end{array}$$ (1)
- Manhattan Distance
  $$\begin{array}{c} d\left(X,Y\right)=|x_{1}-y_{1}\left|+\right|x_{2}-y_{2}\left|+\ldots +\right|x_{n}-y_{n}| \end{array}$$ (2)
- Minkowski Distance
  $$\begin{array}{c} d\left(X,Y\right)=(|x_{1}-y_{1}{|}^{p}+|x_{2}-y_{2}{|}^{p}+\ldots +|x_{n}-y_{n}{{|}^{p})}^{\frac{1}{p}},\;p\in N \end{array}$$ (3)

This distance is the generalization of the two previous ones being the Manhattan distance for *p* = 1, a Euclidian for *p* = 2 and when *p* = ∞ the maximum distance of the modules. In the case when there is more significant information than others, it is possible to include weighting:

$$\begin{array}{c} d\left(X,Y\right)=\sqrt{\omega_{1}{\left(x_{1}-y_{1}\right)}^{2}+\omega_{2}{\left(x_{2}-y_{2}\right)}^{2}+\ldots +\omega_{n}{\left(x_{n}-y_{n}\right)}^{2}} \end{array}$$ (4)

Weights can also be entered into the other two metrics.

KNN is an algorithm that can be used for either classification or regression. It is used in the classification in cases where the attribute is qualitative and used in regression when the attribute is numeric.

The KNN is a classifier that has only one free parameter per stage, the value of *K*. The stage is the classification of an item in the n-dimensional vector of attributes. The value of *K* is controlled by the user in order to obtain a better classification.

The determination of the *K* value is usually carried out by using one of three methods:

- Divination: If you know the problem well, you can have a suggestion;
- Using a heuristic: Avoiding a *K* pair (cases of tie), choosing *K* when it is greater than the number of classes plus one and *K* when it is small enough to avoid noise;
- Using an optimization algorithm: Many algorithms such as genetic or brute- force algorithms can be used, but care must be taken not to increase the value of *K* because it increases the complexity of the classification, and thus makes the algorithm slower.

A KNN classifier with *K* = 1 induces a Voronoi tessellation of the points. This is a partition of space which associates a region *V*(*x*_*i*_) with each point xi in such a way that all points in *V*(*x*_*i*_) are closer to *x*_*i*_ than to any other point. Within each cell, the predicted label is the label of the corresponding training point [38].

A negative aspect of KNN is the so-called Dimensional Curse which is when the classifier does not give very good results for very sparse and large data.

## 4 Measurement campaign

Two measurement campaigns were carried out in the city of Belém-Brazil (1°27’18.62”S, 48°30’08.49”W) throughout 2014, with the objective of acquiring information about the digital TV signal levels in the different scenarios and climatic conditions of the Amazon region.

### 4.1 Description of the climatological features s of the Amazon

The Amazon region has unique climatic conditions and scenarios. It only has two seasons of the year—winter and summer. The Amazonian winter, also known as the rainy season, is characterized by heavy and long-lasting rainfall. The Amazonian summer is characterized by periods of drought and rising temperatures, although sudden rainfall can occur. In addition to the immense Amazonian forest, there are large rivers and large towns and cities in this region. The measurement campaign was carried out in Belém, which is one of the biggest cities located in the Amazon rainforest.

### 4.2 Selection of points and measurement periods

Two measurement campaigns were carried out to analyze possible differences among the signal levels measured under different climatic conditions, during the same year and at the same points. The first campaign was conducted during the months of March and April during the Amazonian winter, and the second campaign occurred in September during the summer of 2014.

Two digital TV stations were selected for this measurement campaign and to provide the next locations and adjacent operating frequencies. TX1 (center frequency 521.14 MHz) and TX2 (center frequency 515.14 MHz) both with a 6 MHz bandwidth. Further information about transmitting antennas can be seen in Table 1.

**Table 1:** Transmitting antenna information.

**Transmitter**	TX1	TX2
**Location**	01°27’43”S/48°29’28”O	01°27’12”S/ 48°29’22”O
**Height (m)**	114.58	125.30
**Band (MHz)**	518-524	512-518
**Transmitted Power (kW)**	6.00	10.00
**Effectively Radiated Power (ERP) (kW)**	52.15	61.79

**Fig 1:**
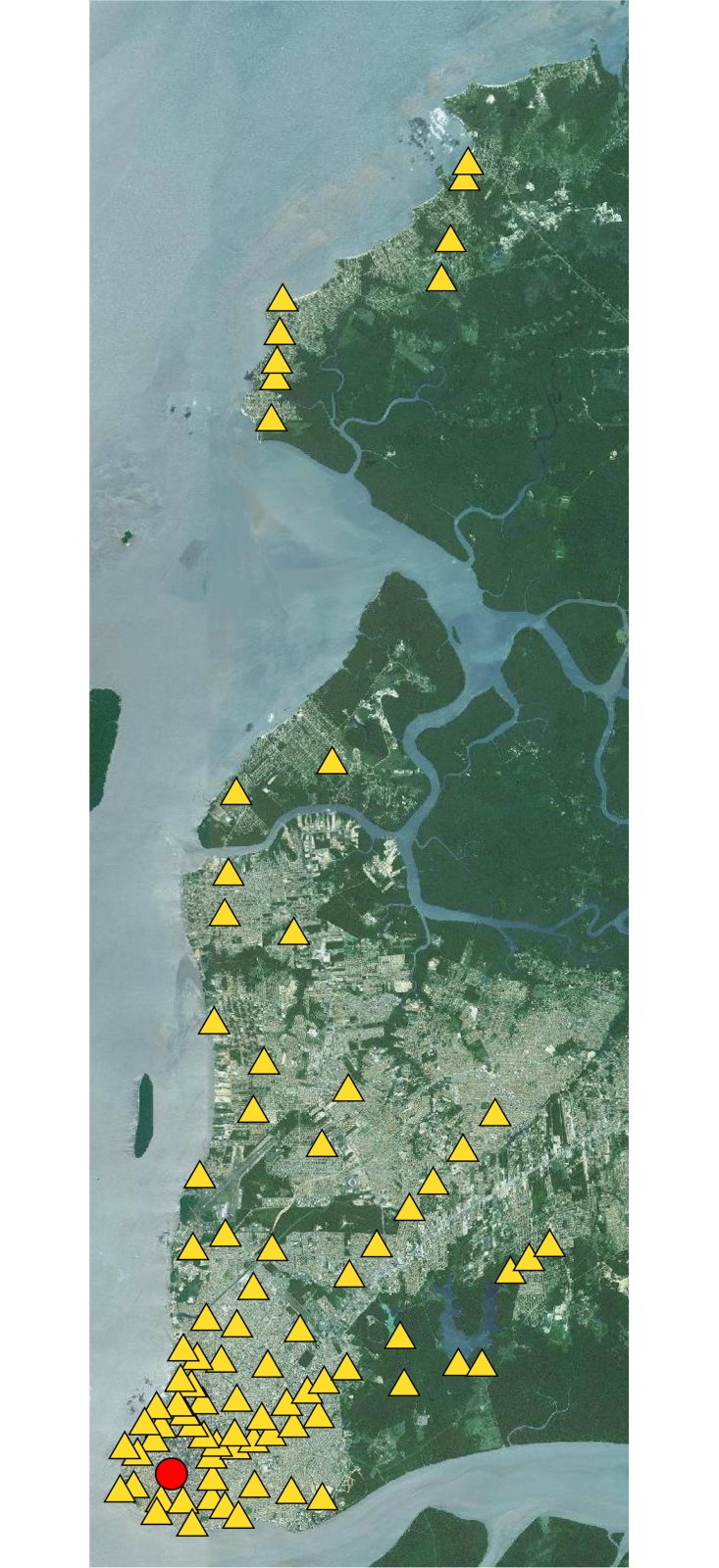
Schamatic map of the city with 14 radials.

The measurements took place at 84 points spread out in 14 radials. The selected points are spread in an area at a minimum distance of 1 *km* and maximum distance of 43 *km* from the *T*_*x*_. Due to the geometrical shape of the city, some radials have more points than others, the shorter radial having just two points. There are different types of terrain in this area, such as urban/suburban paths, Amazon rainforest paths and freshwater paths. Fig 1 shows a schematic map of the city with 14 radials, where the red point is the TX1 and the yellow triangles are the measured points.

### 4.3 Technical devices and measurement setup

The measurement setup consisted of an Anritsu Site Master S332E portable spectrum analyzer, an Anritsu MPP651A dipole antenna for the frequency range between 470 MHz and 1700 MHz and gain of 0 *dB*, a coaxial cable RGC 213, with a characteristic impedance of 50 *ohms* and 3 *m* long.

## 5 Proposed model

The proposed model is a hybrid KNN and KBT algorithm, where initially at each point of the scenario, there is a classification of its attributes, and then, based on this information, a received power value is calculated.

The model was implemented in Matlab^®^. It is empirical and combines qualitative information (building criteria, vegetation, passage through fresh water and season) with quantitative information (location and power), and thus does not have an analytical formulation.

The algorithm performs an interpolation/extrapolation of the bicubic type of the 84 measured points on a grid of 1000 points generated by a 20 × 50 matrix. Its dimensions correspond to the size of the terrain studied in kilometers.

### 5.1 Model inputs

Each measured point of the terrain received six attributes (i.e. a six-component vector). The attributes related to the terrain morphology were given according to what is observed in the proximity of the point. This information was collected locally and from satellite aerial optical images provided by Censipam.

Information regarding the morphology of the terrain was collected locally at the points measured. For distant points of the measured or difficult access (as in the forest) were used aerial optical photos to analyze the morphology.

The qualitative information acquired was arranged into notes, each attribute receiving a different grade in a different range. The terrain morphology attributes are given integer scores in the following ranges: Building [1,5]; Vegetation [1,4]; and Fresh water passage [0,1]. The season attribute is given integer notes [0,1]. Table 2 contains the descriptions and their associated notes.

**Table 2:** Description of attributes and associated notes.

Attribute	Description	Note
Building	Free space	1
	Few houses	2
	Many houses or medium-sized homes	3
	Small buildings	4
	Large buildings	5
Vegetation	No vegetation	1
	Few trees	2
	Many trees	3
	Forest	4
Passing by freshwater	No rivers or lakes	0
	Rivers or lakes	1
Season	Summer	0
	Winter	1
Path loss	Free space equation	-
Estimated power	KNN estimation	-

The quantitative information was incorporated directly into the vector and treated by the generalized KNN regressor. The KNN estimates the power received based on the geographic location of the point and the equation of loss of propagation in the free space described in Eq 5.

$$\frac{P_{T}}{P_{R}}={\left[\frac{4\pi d}{\lambda }\right]}^{2}\frac{1}{G_{T}G_{R}}\frac{1}{F^{2}}$$ (5)

where:

*G*_*T*_—Transmitting antenna power gain;
*G*_*R*_—Gain of receiving antenna power;
*F* = $\frac{E}{E_{0}}$—attenuation factor;
*E*_0_—effective electric field;
*E*—electric field;
λ—wave-length;
*d*—distance from the receiving point to the transmitting antenna.

### 5.2 Techniques used by the proposed model

The proposed model combines three KNNs and two KBTs. The type of input data obtained in the measurement campaigns made this distinction between the types of techniques.

The KNN was chosen because of its dual characteristic as both a classifier and generalized regressor. The KBT was chosen to describe the facts into inference rules and thus be able to infer new information.

The KNN works as a classifier in the analysis of building and vegetation data, by means of the Euclidean distance and *K* = 1, by weaving patterns in the Voronoi diagram for each attribute. It acts as a generalized regressor in the case of the calculation of the power received at any point near a measured point. In both cases, the 84 measured points were used as training points.

The KBT was used in the attributes of the Season and Freshwater passage.

The rule for the winter attribute was based on Dyadic Green’s Function theory described in [38]. In this, a difference of approximately 10 *dB* was determined between the Amazonian summer and winter data.

It was found that there is an increase in the power received after stretches of rivers and lakes, as seen in [8, 9], while in the case of the scenario studied this increase is about 3 dB. Thus, the following rules were drawn up:

- If the season is winter, add 10 *dB* to the calculation of the power received, otherwise add nothing;
- If the point is after fresh water, add 3 *dB* to the calculation of the power received, otherwise add nothing.

### 5.3 Analysis of the types of paths

**Fig 2:**
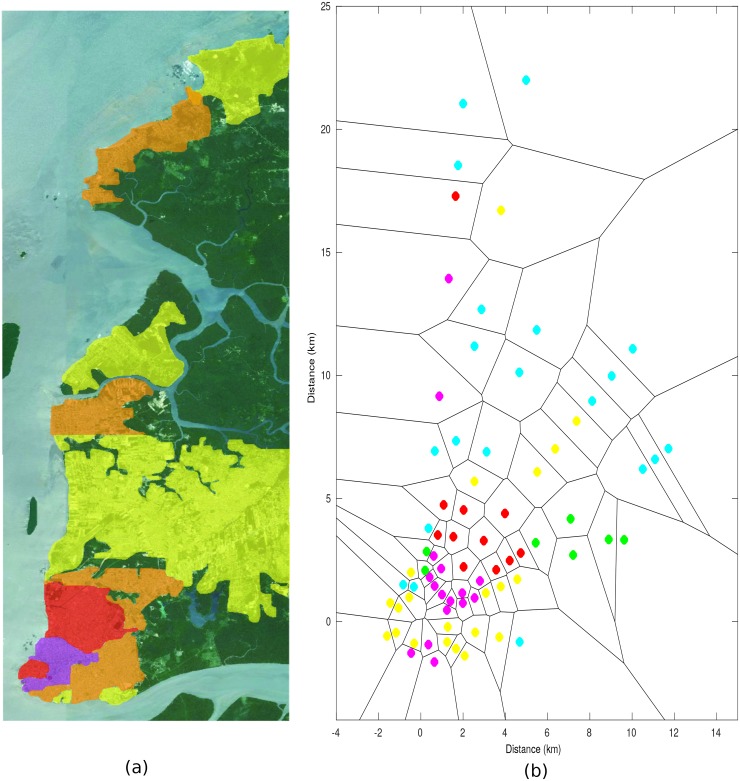
Morphology of the path in accordance with the building criteria, a) Quantification obtained *in loco*, b) Voronoi diagram.

Fig 2(a) shows the quantifications obtained *in loco* of the building attribute. The small patch in purple (upper left corner) is a densely urban area (note 5); the areas in red show many houses and large buildings in a more spaced way (note 4), the orange areas have many medium-sized houses (note 3), the yellow areas have small houses (note 2) and, finally, the green areas do not have any buildings (note 1).

Fig 2(b) illustrates the Voronoi diagram for the building attribute obtained from the model.

**Fig 3:**
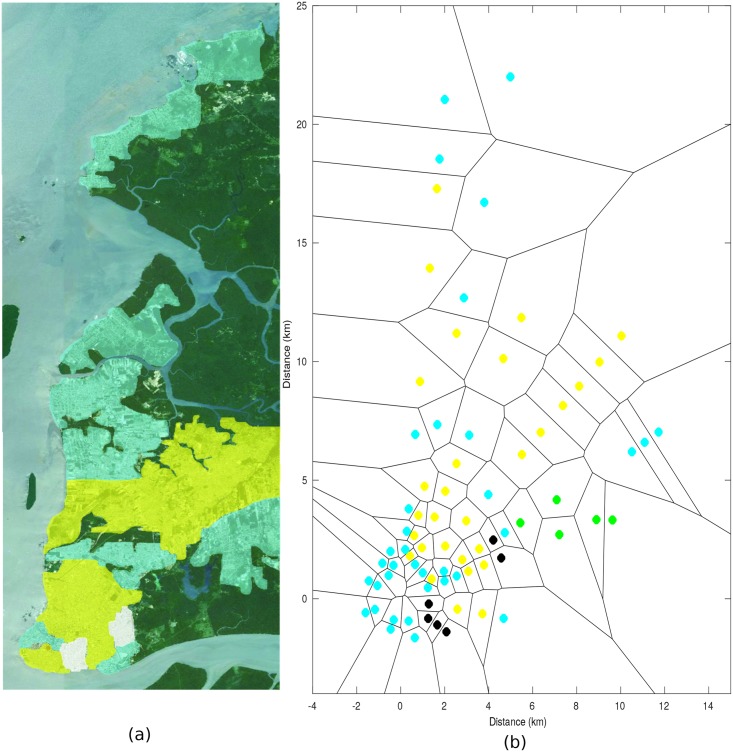
Morphology of the path in accordance with the vegetation criteria, a) Quantification obtained *in loco*, b) Voronoi diagram.

Fig 3(a) illustrates the quantifications of the stretches of land under consideration. The dark green areas are forest regions within the metropolitan area of Belém (note 4). The light blue areas have many trees (note 3); these regions are not limited to suburban areas, but are also present in densely urban areas (compare with the building criteria). The yellow regions have small trees (note 2). Finally, the small white areas are almost non-afforested (note 1). These neighborhoods were unplanned and had many irregular roads and houses in a dilapidated condition and without basic sanitation.

Fig 3(b) illustrates the Voronoi diagram for the vegetation attribute. In this figure, the black spots represent the afforestation attribute with note 1, the other colors are the same as those described for Fig 3(a).

Only two notes were used to classify the stretches of fresh water. Note 1 was attributed to the land after the fresh water and note 0 to the other regions. This means that only two groups of points fall within footnote 1—the radial points 4 that lie behind the extensive lakes of Bolonha and Água Negra (the lakes that supply the city of Belém) and the points of radials 7 and 8 that are located in the district of Mosqueiro beyond the Guajará Bay.

## 6 Results

The model estimates the power received at each of the 1000 points of a surface where the z axis shows the estimated powers.

**Fig 4:**
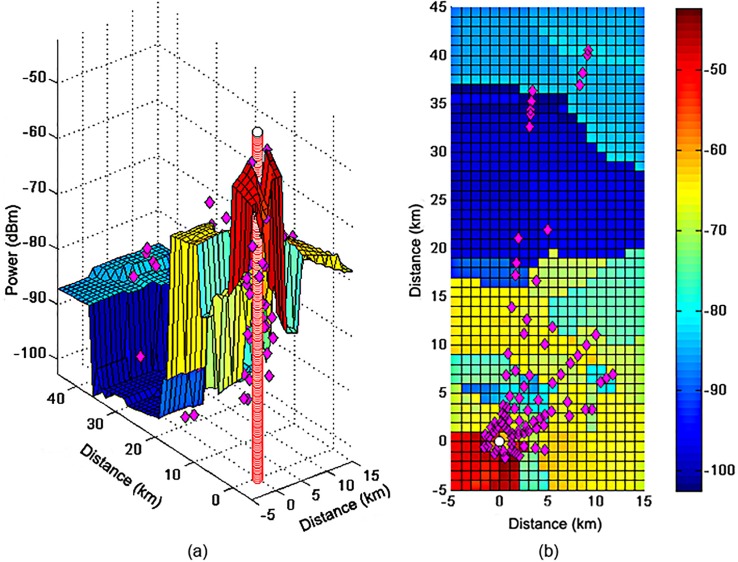
(a) Power surface estimated by the model. (b) Planned surface.

Fig 4(a) and 4(b) show the surface power estimated by the model. The measured powers are represented by a magenta diamond and the transmitter is represented by a red cylinder. These make it possible to observe that the surface follows the behavior of the measured points with higher powers (−50 *dBm*) near TX1 (origin of distances) and the lower powers in the farthest regions (−90 *dBm*), which are the most basic aspects of attenuation as a function of distance.

Fig 4(a) shows a decline at points relatively close to those at TX1 (the green and blue regions); these points are located in densely urban and wooded regions. It was determined by the measurements that the power received is smaller as a function of greater variability. However, in these regions the measured points are even lower, which shows that the model is attempting to follow the behavior, but still needs more adjustments. Near kilometer 20 values of power can be found as low as those that are beyond the Guajará Bay, and around the kilometer 35. Fig 4(b) shows a large yellow area (−65 *dBm*) which is quite far from TX1. Despite the distance from TX1, these areas have a good signal, possibly because they are suburban areas where the main obstructions are trees and not buildings.

### 6.1 Analysis of the path loss profile

**Fig 5:**
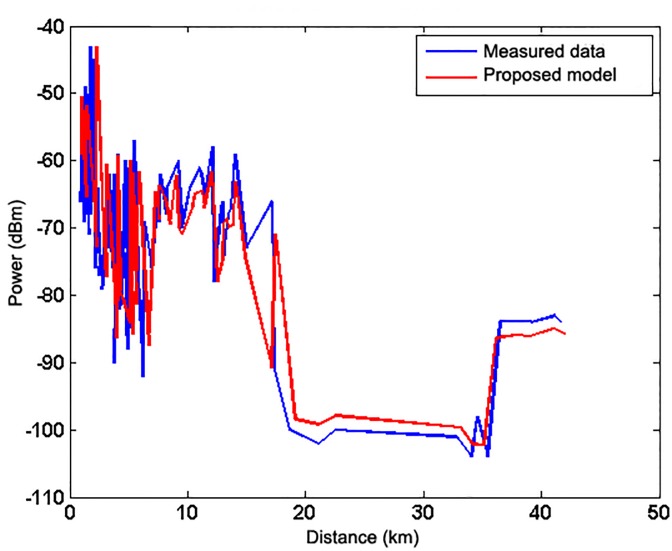
Comparison of the measured data with the model for TX1.

In a linear view, the path loss was calculated by taking the average of the power values received from the internal points to the concentric disks centered on each of the transmitters, as it can be seen in Fig 5.

### 6.2 Results by radial

Another way of analyzing the results is to compare the model with the data measured in the 14 radials. A t-Student test was conducted in each of the radials and in the total set of points of the distances with the powers. In all cases, the null hypothesis test was positive, which means that the averages for the dataset of the model follow the average of the data measured with a 95% confidence interval (standard confidence interval).

In all comparisons the RMS error is calculated from the equation:

$$\begin{array}{c} E_{RMS}=\sqrt{\frac{\sum {\left(X_{M}-X_{E}\right)}^{2}}{n}} \end{array}$$ (6)

where:

*X*_*M*_—measured data;
*X*_*E*_—estimated data;
*n*—number of data.

**Fig 6:**
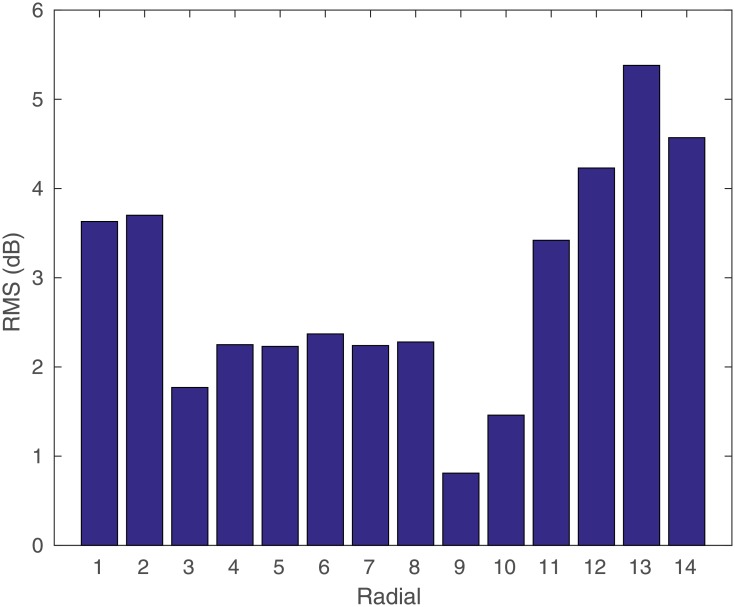
RMS errors for each radial.

The RMS error (in *dB*) among the measured data and the proposed model can be seen in each of the radials in Fig 6. The errors are around 3 *dB*, the smallest being around 0.8 *dB* and the largest 5.5 *dB*.

### 6.3 Results for different climatic conditions

The model distinguishes between weather conditions, by giving different outputs for different climates from information provided by the user. According to the rule established in Section 5, the model predicts a difference of about 10 *dB* between the signal obtained in summer and the signal obtained in winter.

KBT carries out the training with one dataset, but the other sets are unknown to the model. This makes the unknown sets a test, which determines if the model behaves in an expected way.

The model was trained with a set of 84 points measured in the winter for the transmitter TX1. The test data were data sets of the same size, from the two TX1 and TX2 stations for winter and summer, unknown for the model.

**Fig 7:**
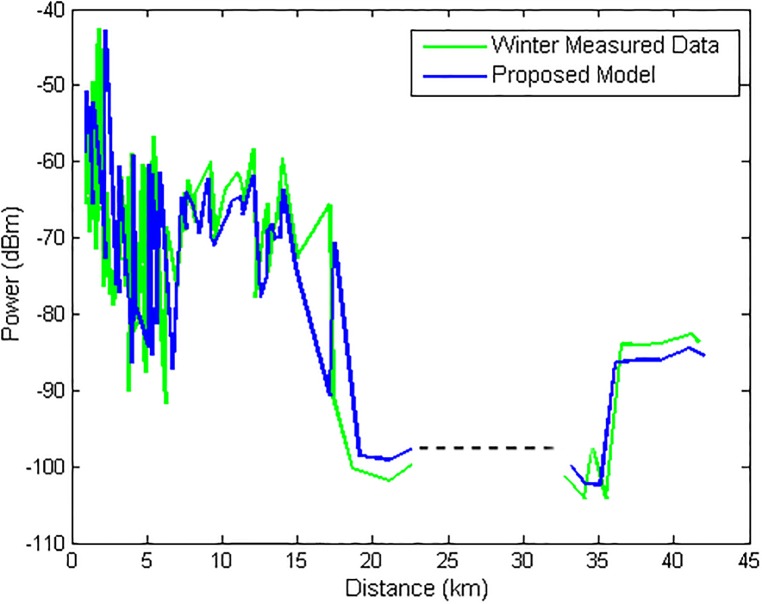
Comparison of the measured data with the model for TX1 for winter.

**Fig 8:**
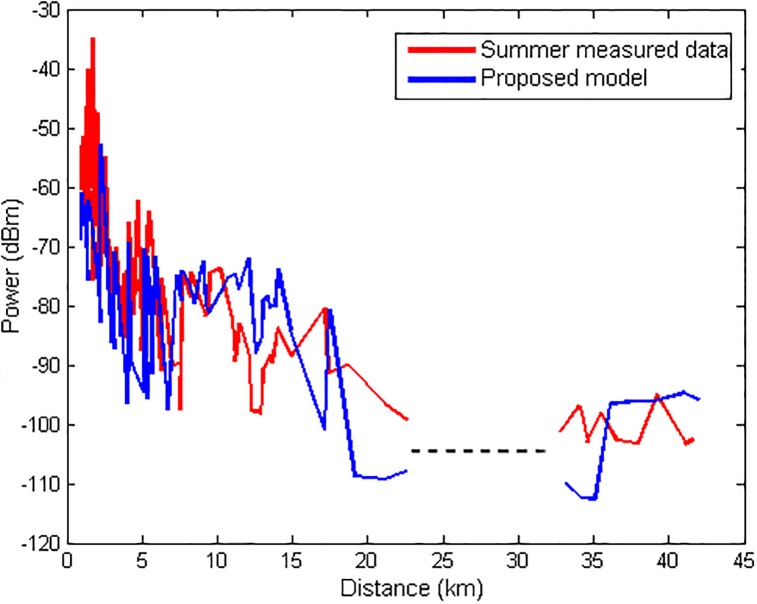
Comparison of the measured data with the model for TX1 for summer.

Figs 7 and 8 illustrate the comparison that is made between the measured data (unknown to the model) and the output of the model output for the winter and summer seasons, respectively. In both figures, there is a gap in the graph that represents the location of Guajará Bay (where no measurements were made). The model showed an RMS error of 1.71 *dB* in the winter and 3.31 *dB* in the summer. The performance difference in the model is due to the fact that it uses a training set with winter data.

## 7 Comparison and validation

The proposed model was compared with three different models, one for each attribute type analyzed. First, it was compared with the Hata model [41] to analyze the building attribute. For the analysis and comparison of the attributes of vegetation and the freshwater passage, the model was compared with the parabolic model [42] and the mixed terrain model [43].

The traditional Hata model is an empirical point-area model for homogeneous paths. Although the Hata model is considered to be a classic model, it remains a basic and important model for making comparisons [43]. For urban area the Hata model is equated by:

$$\begin{array}{c} L_{U}\left(dB\right)=69.55+26.16\log f-13.82\log h_{et}-a\left(h_{er}\right)+\left(44.9-6.55\log h_{et}\right)\log d \end{array}$$ (7)

where:

*L*_*U*_—Path loss in urban areas in decibel (*dB*)
*f*—frequency in MHz;
*h*_*et*_—effective height of the transmitting antenna in meters (*m*);
*h*_*er*_—effective height of the receiving antenna in meters (*m*);
*d*—distance between transmitting and receiving antennas in kilometers (*km*);
*a*(*h*_*er*_)—correction factor for effective height of the receiving antenna which is a function of the size of the area covered.

For a medium-sized city this factor is given by:

$$\begin{array}{c} a\left(h_{er}\right)=\left(1.1\log f-0.7\right)h_{er}-\left(1.56\log f-0.8\right)\;dB \end{array}$$ (8)

For large cities this factor is given by:

$$\begin{array}{c} a\left(h_{er}\right)=\{\begin{array}{c} 8.29{\left(\log\left(1.54h_{er}\right)\right)}^{2}-1.1,\;if\;150\;\leq f\leq 200\\ 3.2{\left(\log\left(11.75h_{er}\right)\right)}^{2}-4.97,\;if\;200\;\leq f\leq 1500 \end{array}\} \end{array}$$ (9)

Path loss for suburban areas:

$$\begin{array}{c} L_{SU}=L_{U}-2{\left(\log\frac{f}{28}\right)}^{2}-5.4 \end{array}$$ (10)

Path loss for rural environments:

$$\begin{array}{c} L_{R}=L_{U}-4.78{\left(\log f\right)}^{2}+18.33f-40.94 \end{array}$$ (11)

To compare the models, the equations of the Hata model were chosen for each type of environment contemplated in the formulation.

**Fig 9:**
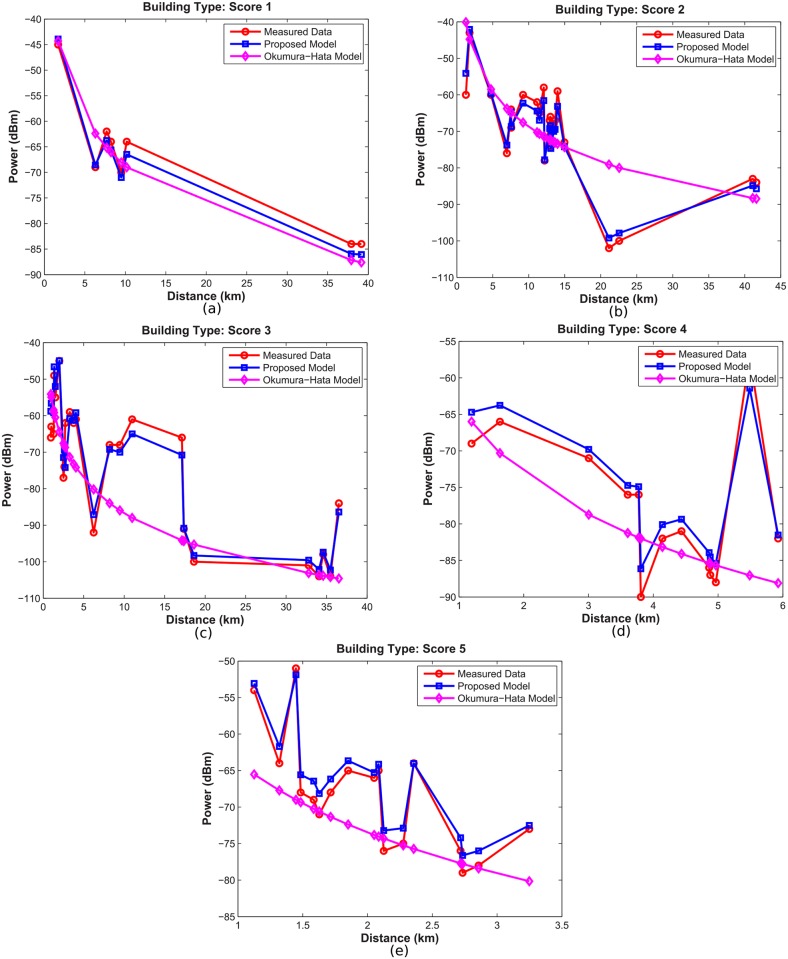
Hata model versus proposed model.

Fig 9 shows the comparison between the proposed model and the Hata model for each type of building. It can be seen that the Hata model follows the average trend of the measured data, but in some paths the difference reached 25 *dBm*. The proposed model achieved a better performance than the Hata model, since it makes an addition to the building attribute, as well as the vegetation attributes and passage through fresh water. A comparison of errors can be seen in Table 3. In all cases, the proposed model showed smaller RMS errors, with errors between 1.67 *dB* and 4.25 *dB*, while the Hata model had errors between 3.71 *dB* and 13.09 *dB*. In both cases the type of building (note 3) had the biggest RMS error, possibly due to the great variability of the data.

**Table 3:** Comparison of RMS errors between the proposed model and the hata model for the different notes for the building attribute.

Type of building	RMS error (dB)	
	Proposed Model	Hata Model
1	1.67	7.19
2	2.41	9.50
3	4.25	13.09
4	2.57	10.12
5	1.87	3.71

**Fig 10:**
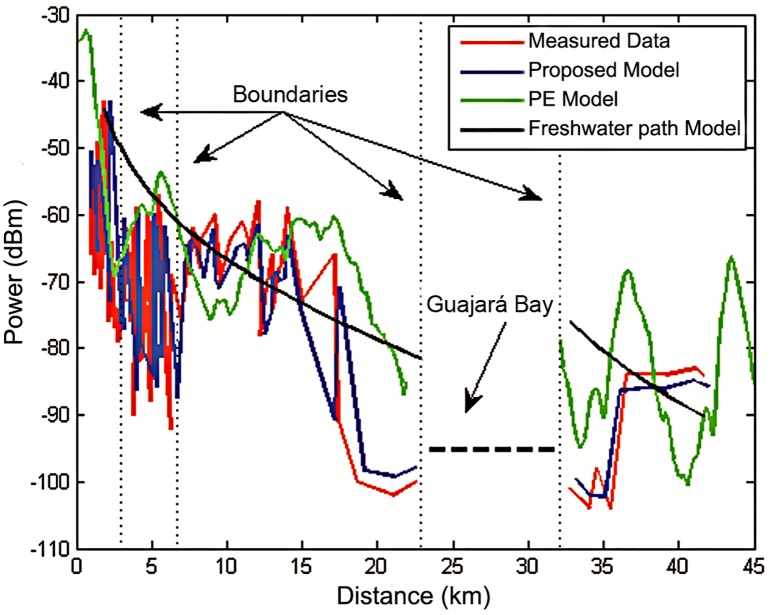
Comparative profile between measured data, the proposed model and parabolic equation model and freshwater path model.

An empirical model based on ITU-R recommendation P.1546-4 is examined in [43]. In this model a correction is made based on the recommendations for a mixed path with fresh water. The model in [42] is a deterministic model that uses partial parabolic differential equations to estimate the power received in a forest environment. Fig 10 shows the comparative profile between the measured data, the proposed model, the freshwater path model [43] and the PE model [42]. The model in [43] is a well-behaved logarithmic model and can make a correction for points beyond the Guajará Bay. However, the proposed model is better suited to the measured data, since it takes account of two other criteria—vegetation and the buildings. The proposed model and the PE model achieved a good performance by following the measured data. In both cases, there was an increase in the power received at the borders of different morphologies (dashed vertical lines) which showed the recovery effect.

Table 4 shows the error comparison for each applied model. Once again, it is evident that the best performance of the proposed model is with an RMS error of 1.68 *dB*, while the mixed path model has a 6.99 *dB* error and the PE model has a 7.61 *dB* error.

**Table 4:** Comparison of errors between the proposed model, freshwater path model and parabolic equation model.

Model	RMS error (dB)
Proposed Model	1.68
Freshwater path Model	6.99
Parabolic equation Model	7.61

## 8 Conclusion

This paper has set out an empirical radio propagation model for DTV for non- homogeneous paths and different climates using machine learning techniques. The model includes several innovative features. The first is the hybrid character of the model, which employs two types of machine learning in the theoretical/computational approach, and thus make s it possible to treat different attributes (quantitative and qualitative) in a clearer and more efficient way. Another innovation is the application of the proposed model for non-homogeneous paths that encompass different scenarios of urbanization and vegetation, including large fresh water paths. The data analysis showed an increase of approximately 3 *dB* in the power received after fresh water, possibly due to the reflection of the signal on the water. These scenarios are common in towns and cities located in equatorial/tropical forests and are rarely addressed in international propagation models and their guidelines.

The distinction between UHF band stations is the culmination of the model, since few studies conduct an analysis of rainfall, wind or humidity for the frequency range under consideration. The measurement campaigns that intentionally took place in both the Amazonian winter and summer and the theory of Dyadic Green’s Functions outlined in [44], established the rule for the seasons, and allowing patterns to be detected of the differentiation between them.

The validation of the model was made by means of a comparison and the model was used to analyze the attributes of vegetation, and mixed terrains, [41–43]. Different Hata model equations were chosen to allow a satisfactory comparison to be made with the building attribute. In all cases, the proposed model showed smaller RMS errors, (between 1.67 *dB* and 4.25 *dB*), while the Hata model had errors between 3.71 *dB* and 13.09 *dB*. The proposed model was able to make good adjustments for points beyond the Guajará Bay by increasing received power from the frontiers of different morphologies, and the recovery effect It had an even lower RMS error of 1.68 *dB* while the model for mixed terrains had an error of 6.99 *dB* and the model of parabolic equations an error of 7.61 *dB*.

It can be concluded from all the factors discussed that the proposed model achieved consistent results and can be applied to other scenarios and climates. In future work, it is intended to make our model a parametric one, for easier implementation.
